# Investigation into the Adhesion Performance of the Recycled Asphalt Mastic–Limestone Filler Interface Using the Pull-Off Test and Surface Free Energy Theory

**DOI:** 10.3390/ma19020394

**Published:** 2026-01-19

**Authors:** Xiaowei Chen, Bailin Wang, Bo Tang, Zhiqian Liang, Xu Lu, Zebang Deng, Bo Li

**Affiliations:** 1 Ningxia Transport Science Research Institute Co., Ltd., Yinchuan 750001, China; 2 Baiyin Highway Development Center of Gansu Province, Baiyin 730900, China; 17794301260@163.com; 3 Gansu Yuanda Road Industry Group Co., Ltd., Lanzhou 730030, China; 4 Gansu Industry Technology Center of Transportation Construction Materials Research and Application, Lanzhou Jiaotong University, Lanzhou 730070, China; dengzebang2001@163.com (Z.D.);

**Keywords:** recycled asphalt mastic, pull-off test, surface free energy, adhesion performance, contribution ratio

## Abstract

This study aims to evaluate the adhesion performance of the recycled asphalt mastic (RAM)–limestone filler interface and the contribution ratio (CR) of mineral filler to the adhesion performance of the RAM–limestone filler interface. Using the pull-off test and surface free energy theory, the impacts of aged asphalt binder dosage, resurfacing dosage, and filler–asphalt binder ratio (F/A) on the grip capacity of the RAM–limestone filler contact were examined. The CR of mineral filler to the adhesion performance of the RAM–limestone filler interface under different influencing factors was calculated. Results show that the bonding strength and adhesion work of the RAM–limestone filler interface reach their best when the aged asphalt binder dosage is 20%. As the rejuvenator dosage increases, the RAM–limestone filler interface’s adhesion performance declines. As the amount of mineral filler increases, the bonding strength of the RAM–limestone filler interface first rises and subsequently falls. When the dosage of aged asphalt binder is 20%, the mineral filler’s CR to the RAM–limestone filler interface’s adhesion performance is at its highest. The impact of mineral filler on the RAM–limestone filler interface’s adhesion performance can be lessened by increasing the amount of rejuvenator. The greatest CR of mineral filler to the adhesion performance of the RAM–limestone filler interface occurs when the F/A is 1.4.

## 1. Introduction

Recycled asphalt (RA) mixtures function as complex dispersion systems consisting of aggregates suspended in RAM. This mastic comprises a blend of mineral fillers, rejuvenators, and both virgin and aged asphalt binders, playing a critical role in bonding, void filling, and load transmission [1]. Within the RA mixture structure, the interface between the RAM and the aggregate is widely recognized as a mechanically weak zone [2]. This interface is particularly susceptible to deterioration caused by external stressors, such as traffic loading and moisture. Moisture-induced damage in RA mixtures typically stems from inadequate bonding forces at this interface, leading to the stripping of the mastic from the aggregate surface [3]. Consequently, accurately evaluating the adhesion capabilities of the RAM–limestone filler interface is fundamental to ensuring the long-term moisture stability of RA mixtures.

As a primary component of RAM, the adhesive properties of the RA itself heavily influence the overall interfacial performance. Yan et al. [4] combined molecular dynamics simulations with SFE analysis, revealing that the adhesion-enhancing effect of rejuvenators on aged binders diminishes with increasing dosage. Similarly, Cao et al. [5] applied SFE analysis to aged and rejuvenated SBS-modified binders, noting that rejuvenators can partially restore adhesion to aggregates. Li et al. [6] observed that waste oil-based RA offers superior moisture resistance despite a 55% drop in adhesion work. Furthermore, Li et al. [7] employed the sessile drop method to calculate adhesion and peeling work, concluding that increasing the aged binder content results in a decrease in the system’s adhesion work. Collectively, these studies highlight that adhesion is significantly affected by the rejuvenator and the state of the asphalt binder. However, the incorporation of mineral filler to form RAM further alters these properties. Therefore, greater attention must be directed toward the adhesion performance of the RAM–limestone filler interface.

Extensive investigations have been carried out regarding the adhesive properties of standard asphalt mastic–aggregate interfaces [8]. Given the similarities, these established methodologies provide a valuable framework for evaluating RAM–limestone filler interfaces under various conditions. The pull-off test is frequently employed for such assessments. For instance, Lei et al. [9] manufactured mastic with recycled concrete powder and used pull-off tests to demonstrate that higher F/A ratios compromise bond strength. Zhang et al. [10] used a PosiTest AT-A tester to evaluate red mud asphalt mastics, finding that red mud negatively impacts interfacial adhesion. Conversely, Ma et al. [11] combined pull-off testing with gray correlation analysis on water-conditioned samples, suggesting that additives like hydrated lime and cement improve mastic adhesion. While these studies utilize macro-scale pull-off tests, employing a diverse range of testing methods is essential for a more precise, quantitative assessment of the RAM–limestone filler interface.

Surface free energy (SFE) theory is another methodology frequently applied to analyze the adhesion of asphalt mastic–aggregate interfaces [12,13]. Zhang et al. [14] found that porous fillers with high metal content increase mastic polarity, thereby enhancing moisture resistance. In a study concerning salt storage additives, Xu et al. [15] used SFE to demonstrate that increasing salt storage additive content leads to higher contact angles and slightly lower mastic SFE, indicating reduced wettability. Additionally, Zhang et al. [16] elucidated the moisture damage mechanism from the perspective of SFE theory, categorizing the interaction between mineral fillers and asphalt into four distinct mechanisms: ‘physical hardening’, ‘chemical bonding’, ‘water instability’, and ‘polarity enhancement’.

To summarize, methodologies grounded in pull-off testing and surface free energy theory have become standard for evaluating asphalt mastic adhesion. Nevertheless, while general RAM performance is well-studied, there remains a scarcity of research specifically targeting the quantitative contribution ratio of mineral fillers to the adhesion of the RAM-aggregate interface. Furthermore, existing studies on asphalt mastic interfaces primarily focus on the impact of F/A ratios, often overlooking other critical variables. Consequently, this study aims to clarify the influence of mineral fillers on the RAM–limestone filler interface by addressing three specific objectives. First, the investigation analyzes the impact of aged binder and rejuvenator dosages on interfacial bonding strength and surface energy. Second, the work determines the optimal F/A to maximize adhesion. Third, the study quantifies the specific CR of the mineral filler to the total adhesion performance.

**Figure 1:**
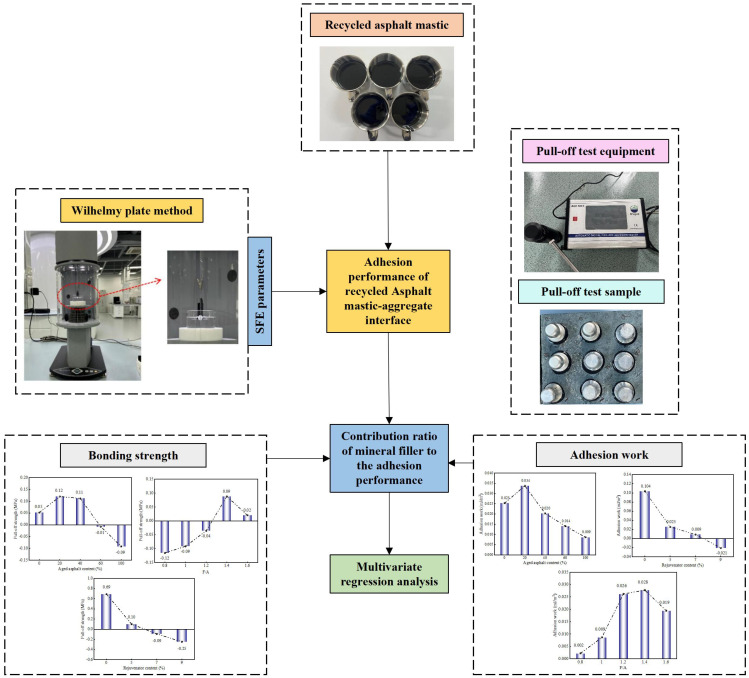
Flowchart of the experimental design.

This paper employs the pull-off test to examine how variations in aged binder content, rejuvenator levels, and F/A ratios impact the adhesion of the RAM–limestone filler interface. Simultaneously, the SFE parameters of the RAM were determined using the Wilhelmy plate method to facilitate an analysis based on SFE theory. A key contribution of this work is the calculation of the CR of mineral fillers to the interfacial adhesion performance under these differing factors. The experimental design and workflow are illustrated in Figure 1.

## 2. Materials and Experimental Methods

### 2.1. Raw Materials

A virgin asphalt binder, classified with a penetration grade of 80–100 mm, served as the base material for this study. The essential physical parameters characterizing this binder are presented in Table 1.

A laboratory-synthesized rejuvenator was developed specifically for this investigation. The rejuvenator is a laboratory-prepared composite blend formulated to restore the colloidal balance of the aged binder. It consists of waste bio-oil as the base regenerant and a plasticizer to adjust viscosity, combined with a PS toughening agent and ground C5 petroleum resin to enhance ductility and adhesion properties. Its performance characteristics were evaluated in full alignment with the Technical Specifications for Highway Asphalt Pavement Recycling (JTG/T 5521-2019) [17]. The resulting physical data for this additive are presented in Table 2.

Limestone mineral filler was selected as the particulate component for the RAM. Its physical specifications are listed in Table 3.

### 2.2. Specimen Fabrication

#### 2.2.1. Asphalt Binder Preparation

(1) Simulation of aged binder

Recognizing the operational complexities and limited availability of Pressure Aging Vessel (PAV) equipment, long-term aging was simulated by extending the duration of the Rolling Thin Film Oven Test (RTFOT) [18,19]. The procedure involved heating the binder to a fluid state at 135 °C, transferring 35 ± 0.5 g into aging bottles, and subjecting them to rotating film heating for a duration of 270 min. This process yielded the simulated PAV-aged binder specimens.

(2) RAM formulation

The specific compositions of the RAM variants are outlined in Table 4, with dosages selected based on a threefold rationale: a 20% aged binder content was chosen to simulate a representative surface layer recycling scenario; a 7% rejuvenator dosage was empirically determined to restore the aged binder’s viscosity to the rheological target of the virgin binder; and an F/A range of 0.8–1.6 was selected to bridge standard specification limits with filler-rich conditions [13]. For specimen preparation, the limestone filler was pre-conditioned at 105 °C for 4 h, while the binder phase—comprising virgin binder, aged binder, and rejuvenator—was blended at 135 °C under a shear speed of 2000 r/min for 5 min. Subsequently, the filler was incrementally incorporated at the designated ratios, followed by 20 min of continuous stirring to guarantee homogeneity and effective deaeration, after which the final RAM samples were sealed and stored at ambient temperature to maintain integrity prior to testing.

#### 2.2.2. Test Specimen Preparation

(1) Pull-off test samples

The film thickness of the RAM is a decisive factor governing adhesion results in pull-off tests. In this study, a standardized thickness of 0.2 mm was maintained, with a tensile loading rate of 0.7 MPa/s [20]. The required mass of RAM was computed based on its density and the target volume. The heated mastic was then cast into 8 mm diameter molds. To ensure sample integrity without deformation, a rapid freeze-demolding process was employed solely for removing the specimen from the mold, which does not affect the material’s fundamental properties.

(2) Contact angle test sample

Glass microscope slides (50 mm × 24 mm × 0.15 mm) were utilized as substrates. The slides were vertically immersed in the molten RAM and immediately inverted to facilitate the natural drainage of excess material, thereby achieving a uniform coating. These coated slides were conditioned in a desiccating oven for 24 h prior to measurements.

### 2.3. Theoretical Framework and Testing Methods

#### 2.3.1. Pull-Off Adhesion Test

Interfacial bond strength was quantified using a BGD 500/S automatic adhesion tester (BIUGED, Guangzhou, China). The instrument’s metal spindle was bonded to the test surface, applying a tensile force perpendicular to the interface. Loading was applied at a constant rate of 0.7 MPa/s. The peak stress recorded at the point of detachment from the aggregate surface was defined as the interfacial bonding strength.

#### 2.3.2. Surface Free Energy (SFE) Principles

The SFE refers to the work to be performed by the outside world when the system increases the unit surface area [20,21]. The SFE can be calculated as follows:

(1) $$\gamma =\gamma^{LW}+\gamma^{AB}=\gamma^{LW}+2\sqrt{\gamma^{+}\gamma^{-}}$$

where γ is the SFE, $\gamma^{LW}$ is the dispersion component, $\gamma^{AB}$ is the polar component, $\gamma^{+}$ is the Lewis acid component, and $\gamma^{-}$ is the Lewis base component.

To determine the surface energy parameters of the RAM, the Young–Dupré relationship, presented in Equation (2), was applied, utilizing the contact angle (*θ*) resulting from the interaction between the mastic substrate and the test liquid:

(2) $$\gamma_{L}(1+\cos\theta )=2\sqrt{\gamma_{L}^{LW}\gamma_{s}^{LW}}+2\sqrt{\gamma_{L}^{+}\gamma_{s}^{-}}+2\sqrt{\gamma_{s}^{+}\gamma_{L}^{-}}$$

According to the van Oss–Chaudhury–Good acid–base theory, the surface free energy comprises three unknown components ($\gamma_{L}^{LW}$, $\gamma_{s}^{+}$, $\gamma_{L}^{-}$). Therefore, contact angle measurements using a minimum of three probe liquids with known parameters are mathematically required to establish a system of three linear equations to solve for these unknowns. Accordingly, this study employed distilled water, glycerol, and formamide as the test fluids, whose respective SFE parameters are cataloged in Table 5. These three specific liquids were selected because they possess distinct polarity and dispersive components that are mathematically sufficient to solve the three unknowns in the acid–base theory equation. Consequently, additional polar liquids were deemed unnecessary, as this trio avoids potential surface dissolution issues while ensuring the robustness of the calculated surface energy parameters.

Upon determining the constituent SFE parameters for both the RAM and the aggregate, the interfacial adhesion characteristics can be quantitatively assessed. This evaluation entails the calculation of the RAM’s internal cohesion work, as well as the adhesion and peeling work associated with the interface. The reference SFE components for the aggregate material are provided in Table 6.

The cohesion work (W_co_), adhesion work (W_as_), and peeling work (W_asw_) can be used to evaluate the adhesion performance between the RAM and the aggregate, as shown in Equations (3)–(5):

(3) $$W_{co}=2\gamma_{a}$$

(4) $$W_{as}=2\sqrt{\gamma_{a}^{LW}\gamma_{s}^{LW}}+2\sqrt{\gamma_{a}^{+}\gamma_{s}^{-}}+2\sqrt{\gamma_{s}^{+}\gamma_{a}^{-}}$$

(5) $$\begin{array}{c} W_{asw}=2\gamma_{w}+2\sqrt{\gamma_{a}^{LW}\gamma_{s}^{LW}}+2\sqrt{\gamma_{a}^{+}\gamma_{s}^{-}}+2\sqrt{\gamma_{a}^{-}\gamma_{s}^{+}}-2\sqrt{\gamma_{a}^{LW}\gamma_{w}^{LW}}-2\sqrt{\gamma_{a}^{+}\gamma_{w}^{-}}\\ \;\;\;\;\;\;\;\;\;\;-2\sqrt{\gamma_{a}^{-}\gamma_{w}^{+}}-2\sqrt{\gamma_{s}^{LW}\gamma_{w}^{LW}}-2\sqrt{\gamma_{s}^{+}\gamma_{w}^{-}}-2\sqrt{\gamma_{s}^{-}\gamma_{w}^{+}} \end{array}$$

where $\gamma_{a}$ is the SFE of RAM, $\gamma_{s}$ is the SFE of aggregate, and $\gamma_{w}$ is the SFE of water.

#### 2.3.3. Wilhelmy Plate Technique

The dynamic contact angle of the RAM was assessed using the Wilhelmy plate method on a Sigma automatic surface tension meter (Biolin Scientific, Shanghai, China) [22,23,24]. The simplified procedure involved immersing the coated glass plate in the probe liquid at a velocity of 2 mm/min to a depth of 8 mm. The resulting force variation (ΔF) was recorded to compute the contact angle via Equation (6):

(6) $$\cos\theta =\frac{\Delta F+V_{im}(\rho_{L}-\rho_{air})g}{P_{t}\gamma_{L}}$$

where *V_im_* denotes the volume of the specimen submerged in the fluid, while *ρ_L_* and *ρ_air_* represent the respective densities of the liquid and the ambient air. Furthermore, *g* is the gravitational acceleration constant, *P_t_* indicates the wetted perimeter of the coated plate, and γ_L_ corresponds to the surface free energy of the test liquid.

## 3. Results and Discussion

### 3.1. Analysis of Pull-Off Test Data

#### 3.1.1. Bonding Strength of RAM with Different Dosages of Aged Asphalt Binder

**Figure 2:**
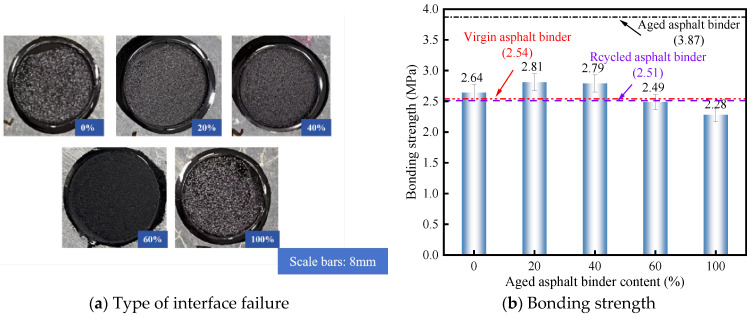
Bonding strength of RAM with different-aged asphalt binder.

**Figure 3:**
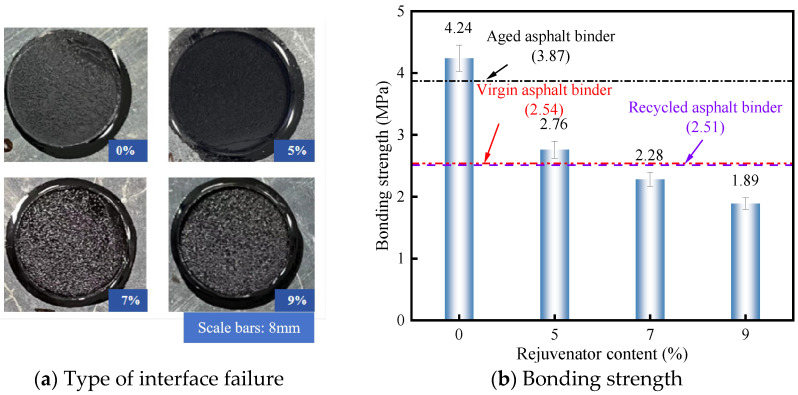
Bonding strength of RAM with different rejuvenators.

**Figure 4:**
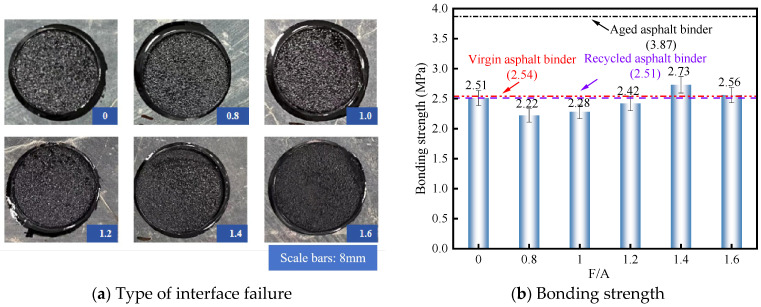
Bonding strength of RAM with different F/A.

There are three types of bond failure in RAM–limestone filler interface: cohesion failure, adhesion failure, and mix failure. Cohesion failure occurs inside the RAM, and the bonding strength reflects the cohesion of the RAM itself. Adhesion failure occurs at the interface between RAM and aggregate, and the bonding strength reflects the adhesion between RAM and aggregate. Mix failure had both adhesion failure and cohesion failure, and the bonding strength is the comprehensive embodiment of adhesion and cohesion. To quantify the failure mode, a visual classification method was employed. The fracture surfaces were inspected after the pull-off test. As shown in Figure 2a, the aggregate surface remained covered by asphalt mastic in over 95% of the visible area. This dominant coverage indicates that the adhesive strength at the interface exceeded the internal cohesive strength of the mastic, confirming a cohesive failure mode. This classification was consistently observed across the test groups. To ensure the statistical reliability of the test results, three parallel samples were tested for each experimental condition. Given the consistency of the automated pull-off tester, a typical coefficient of variation (CV) of 5% was observed across the dataset. Consequently, error bars representing a standard deviation corresponding to this 5% variability have been included in Figure 2, Figure 3 and Figure 4 to illustrate the dispersion of the bonding strength data.

According to the data illustrated in Figure 2b, the reference bonding strength for the RA–aggregate interface was established at 2.51 MPa. Upon the incorporation of mineral filler, the PAV + 20% formulation yielded the optimal performance, surpassing the RA baseline by a margin of 11.9%. A detailed examination reveals that at aged binder concentrations of 20% and 40%, the interfacial bonding strengths were elevated by 0.30 MPa and 0.28 MPa, respectively, relative to the RA control. This enhancement serves as evidence that the mineral filler reinforces the internal cohesion of the RAM under these specific conditions. In contrast, when the aged binder content was increased to 60% and 100%, the bonding strengths dropped to 2.49 MPa and 2.28 MPa, respectively. These values fall below the RA–aggregate benchmark, implying that in the presence of high aged binder concentrations, the mineral filler exerts a detrimental influence on the mastic’s cohesive integrity.

#### 3.1.2. Bonding Strength of RAM with Different Rejuvenator Dosage

The influence of rejuvenator dosage on bonding strength is presented in Figure 3. Figure 3a confirms that cohesive failure remains the dominant failure mechanism regardless of rejuvenator content. From Figure 3b, it is evident that a 5% rejuvenator dosage yields a bonding strength of 2.76 MPa, representing a 9.9% improvement over the RA baseline. Conversely, increasing the dosage to 7% and 9% resulted in strength reductions of 9.2% and 24.7%, respectively. These results suggest a competitive mechanism: at low dosages, the filler’s reinforcing effect dominates. However, as rejuvenator content increases, it replenishes light components and softens the mastic, thereby overriding the filler’s benefits and reducing overall cohesive strength.

#### 3.1.3. Bonding Strength of RAM with Different F/A

Figure 4 illustrates the variations in interfacial bonding strength across a range of F/A ratios. As depicted in Figure 4a, the predominant failure mechanism observed under all tested F/A conditions was cohesive fracture, characterized by the mastic remaining firmly attached to the aggregate substrate. Quantitative analysis from Figure 4b reveals that at lower F/A ratios of 0.8, 1.0, and 1.2, the interfacial bonding strength fell below the RA baseline, registering reductions of 11.6%, 9.2%, and 3.6%, respectively. These findings suggest that the filler’s capacity to reinforce mastic cohesion is strictly governed by the specific F/A ratio. At lower filler concentrations, the insufficient interaction surface area results in an excess of free asphalt binder, which consequently diminishes the overall structural integrity of the RAM. Conversely, the bonding strength peaked at an F/A ratio of 1.4, exhibiting an 8.8% improvement over the RA–aggregate interface. This enhancement is attributed to the increased presence of mineral filler, which promotes the formation of a higher proportion of structural asphalt binder within the system, thereby augmenting the intrinsic strength of the mastic.

In addition, when the F/A is 1.6, the bonding strength of the RAM–limestone filler interface gradually decreases, indicating that the mineral filler will not unconditionally increase the cohesion inside the RAM. When the mineral filler dosage reaches a certain threshold, the total area of the interface increases, and the structural asphalt binder formed on the surface of the mineral filler particles becomes thinner. The higher the mineral filler content, the more severe the particle agglomeration. This greatly affects the dispersion state of the mineral filler in the asphalt binder, resulting in uneven stress of the RAM and a decrease in the bonding strength. In summary, the cohesion performance of RAM is affected by the dosage of mineral filler. In the design process of the RA mixture, it is very important to select the appropriate F/A.

### 3.2. Evaluation Based on SFE Theory

#### 3.2.1. SFE Parameter Analysis

The wettability characteristics of the RAM were quantified via contact angle measurements [25,26], with the specific results for various test liquids summarized in Table 7. An analysis of the data reveals that the virgin asphalt binder exhibits a contact angle of 91.38° when tested with distilled water. Conversely, the aged binder displayed a reduction of 3.63° relative to the virgin sample, a trend that underscores the enhanced hydrophilicity induced by the oxidative aging process [27]. Furthermore, the incorporation of mineral fillers precipitated a notable shift in surface properties; specifically, the contact angles for virgin asphalt mastic, aged asphalt mastic, and RAM increased by 5.08°, 4.11°, and 0.58°, respectively, compared to their binder or RA baselines. These findings suggest that mineral fillers contribute to a moderate increase in the hydrophobicity of the mastic system. This modification is presumably driven by the alteration of the surface composition following the integration of the filler into the asphalt binder matrix.

For aged mastic, adding rejuvenator progressively increased the contact angle, suggesting effective restoration of hydrophobic properties. In the case of RA, the contact angle initially rose with filler addition but subsequently decreased at higher F/A ratios, pointing to a limit in the filler’s ability to improve hydrophobicity.

A contact angle of 91.86° was recorded for the aged asphalt mastic when tested against water. Upon increasing the concentration of the rejuvenator, a progressive elevation in the contact angle between the RAM and water was observed. This trend serves as evidence that the inclusion of rejuvenators is effective in mitigating the hydrophilicity associated with aged asphalt mastic. In the case of the RA baseline, a contact angle of 95.16° was established. Following the introduction of mineral filler, the contact angle trajectory initially ascended before exhibiting a decline. This pattern implies that while minor additions of mineral filler contribute to enhancing the hydrophobicity of the RAM, this effect is non-linear. Specifically, as the F/A ratio continues to rise, the presence of excess mineral filler appears to compromise the hydrophobic characteristics of the RAM.

Table 8 details the specific SFE components for the various RAM formulations. An examination of the data reveals that the aged asphalt binder possesses a polar component of 15.50 mJ/m^2,^ representing an elevation of 7.69 mJ/m^2^ relative to the virgin binder baseline. This phenomenon is primarily attributed to the progressive volatilization of lighter fractions throughout the aging cycle. Consequently, the concentration of macromolecular entities and robust polar functional groups rises, thereby intensifying the overall polarity of the aged binder [16]. For the RA itself, the dispersive and polar components were quantified at 3.88 mJ/m^2^ and 11.13 mJ/m^2,^ respectively. Following the incorporation of mineral fillers, a distinct trend emerged where the RAM’s dispersive component exhibited a gradual ascent, whereas the polar component demonstrated a consistent decline. Notably, at an F/A ratio of 1.6, the polar component experienced a substantial reduction of 28.9%. This reduction can be mechanistically explained by the adsorption of polar asphalt molecules onto the filler surface. Specifically, asphaltenes and resins serve as surface-active agents rich in polar constituents such as asphaltic acids and anhydrides, with the carboxyl moiety functioning as a highly reactive polar center. Limestone is primarily composed of CaCO_3_ and acts as an active base that chemically interacts with the acidic functional groups present in the aged asphalt. This chemical bonding mechanism reduces the free polar groups available on the RAM surface, thereby increasing hydrophobicity. These observations align with findings by Zhang et al. [16], who noted that mineral fillers significantly alter the mastic surface energy components through physical adsorption, although the specific magnitude varies with filler type.

#### 3.2.2. SFE of RAM with Different Dosages of Aged Asphalt Binder

**Figure 5:**
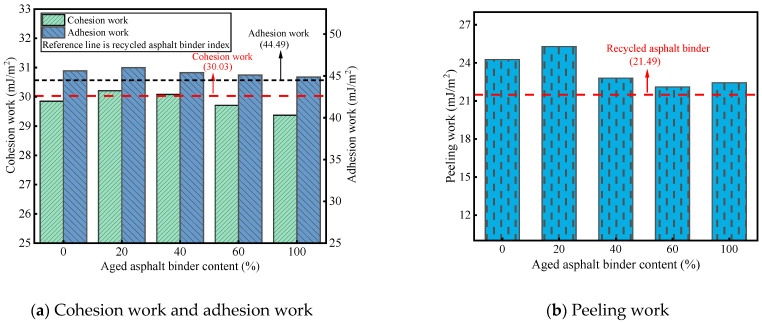
Adhesion index of RAM with different-aged asphalt binders.

Figure 5 illustrates the variations in adhesion parameters between the RAM and aggregate across different concentrations of aged asphalt binder. As depicted in Figure 5a, the cohesive energy density of the RAM consistently falls below the interfacial adhesion work values. This observation serves as further corroboration for the failure modes identified in the pull-off tests, where cohesive failure within the mastic was predominant. Specifically, the cohesion work for the reference RA stands at 30.03 mJ/m^2^. Upon the incorporation of mineral fillers, the RAM’s cohesion work exhibits a non-linear trend, initially ascending before subsequently declining. Notably, samples containing 20% and 40% aged binder demonstrated cohesion values superior to those of the RA. The metric peaked in the PAV + 20% specimen, which registered a value 0.18 mJ/m^2^ higher than the RA baseline.

Regarding the interfacial adhesion work, the RAM–limestone filler system consistently outperformed the RA–aggregate, regardless of the aged binder concentration. However, a similar trend of initial improvement followed by a reduction was observed. The peak adhesion work was also recorded at an aged binder content of 20%, exceeding the RA–aggregate interface by 0.38 mJ/m^2^. These findings underscore that the inclusion of mineral fillers effectively enhances the interfacial bonding performance.

Figure 5b presents the peeling work data. The lowest peeling work was observed at an aged binder dosage of 60%, 22.10 mJ/m^2^; however, this value remained higher than that of the RA–aggregate interface. Furthermore, at aged binder concentrations of 40%, 60%, and 100%, the interfacial peeling work exceeded that of the virgin asphalt mastic. This suggests that a higher proportion of aged binder may, to a certain degree, augment the moisture stability of the interface.

#### 3.2.3. SFE of RAM with Different Dosages of Rejuvenator

**Figure 6:**
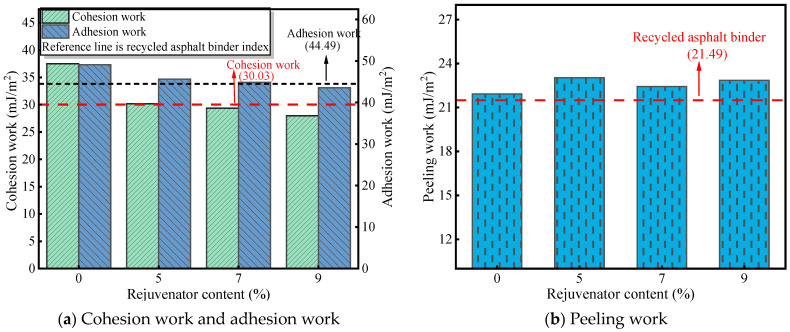
Adhesion indices of RAM with different rejuvenator dosages.

The impact of rejuvenator dosage on the RAM–limestone filler adhesion metrics is detailed in Figure 6. Referring to Figure 6a, the aged asphalt mastic exhibits cohesion and adhesion work values of 37.49 mJ/m^2^ and 49.1 mJ/m^2^, respectively. These figures represent significant increases of 7.64 mJ/m^2^ and 3.49 mJ/m^2^ over the virgin asphalt mastic. This enhancement is primarily attributed to the aging process, which involves the volatilization of lighter fractions and a relative increase in heavy components, thereby augmenting the cohesive strength of the binder itself and its adhesion to the aggregate.

When the rejuvenator concentration is set at 5%, the RAM displays a cohesion work of 30.16 mJ/m^2^, marginally surpassing that of the RA. However, as the quantity of rejuvenator increases, a gradual reduction in RAM cohesion work is observed, a trend that aligns with the bonding strength results from the pull-off tests. In comparison to the RA–aggregate interface, the RAM samples with 5% and 7% rejuvenator showed improved adhesion work, increasing by 1.13 mJ/m^2^ and 0.38 mJ/m^2^, respectively. Conversely, at a 9% dosage, the adhesion work dropped by 0.93 mJ/m^2^. This indicates that while mineral fillers can bolster adhesion at lower rejuvenator levels, excessive rejuvenator content may disrupt the colloidal structure by introducing too many light components, thereby limiting the filler’s efficacy [4].

As shown in Figure 6b, the peeling work for the RAM–limestone filler interface is generally lower than that of the RA baseline. Although an abrupt change occurs at the 5% dosage level, the overall trajectory is upward. This implies that increasing the rejuvenator content acts detrimentally to the interface’s adhesion when moisture is present.

#### 3.2.4. SFE of RAM with Different F/A

The adhesion work characterizing the RAM–limestone filler interface consistently surpasses that of the RA–aggregate baseline, following a non-linear trajectory that initially ascends before declining as the F/A ratio rises. The optimal adhesion performance was identified at an F/A ratio of 1.4, where the adhesion work achieved a peak value exceeding the RA–aggregate interface by 1.23 mJ/m^2^. Conversely, extending the F/A ratio to 1.6 precipitated a reduction in adhesion work. These observations demonstrate that while the incorporation of mineral fillers is beneficial for reinforcing interfacial adhesion, this enhancement is dosage-dependent; excessive filler content eventually compromises the adhesive integrity of the RAM–limestone filler system.

**Figure 7:**
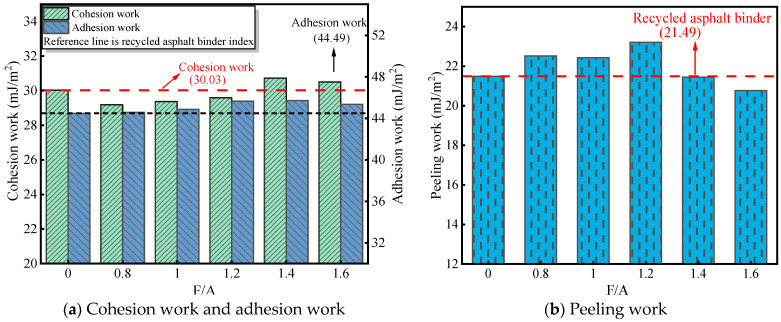
Adhesion indices of RAM with different F/A ratios.

As shown in Figure 7b, the peeling work of the RA–aggregate interface is 21.49 mJ/m^2^. When the F/A is 0.8, 1.0, and 1.2, the peeling work of RAM–limestone filler interface increases by 4.6%, 4.4%, and 8.0%, respectively. When the F/A is 1.4 and 1.6, the peeling work of the RAM–limestone filler interface is lower than that of the RA–aggregate interface, which is reduced by 0.04 mJ/m^2^ and 0.72 mJ/m^2^. It shows that under the condition of water, high dosage of mineral filler helps to improve the adhesion performance of RAM–limestone filler interface and has the effect of preventing RAM from falling off from the aggregate surface.

Collectively, a comparative analysis between the mechanical pull-off tests in Section 3.1 and the thermodynamic SFE results in Section 3.2 reveals a strong correlation regarding the optimal F/A ratio of 1.4 and aged binder dosage of 20%. Both methods confirm that moderate filler addition reinforces the interface. However, a notable divergence is observed regarding rejuvenator dosage. While SFE calculations suggest improved wettability at higher dosages, as shown in Figure 6b, mechanical bonding strength declines as illustrated in Figure 3b. This discrepancy highlights that SFE characterizes the potential thermodynamic adhesion, whereas the pull-off test reflects the actual mechanical strength, which is heavily governed by the bulk cohesion of the mastic. Practically, this implies that although rejuvenators restore surface wettability, excessive usage compromises the cohesive integrity of the mastic and leads to premature failure under mechanical load. Therefore, engineering designs must balance thermodynamic wetting potential with macroscopic cohesive strength.

### 3.3. The CR of Mineral Filler to the Adhesion Performance

#### 3.3.1. The CR of Mineral Filler to the Bonding Strength

The impact exerted by mineral fillers on the adhesive integrity of the RAM–limestone filler interface is highly variable, contingent upon specific parameters such as the content of aged asphalt binder, the concentration of rejuvenator, and the F/A ratio. To quantitatively assess the specific role of the mineral filler under these diverse conditions, a specialized metric known as the CR index was developed. The CR index was developed to normalize the enhancement effect of the mineral filler against the baseline performance of the asphalt binder. By subtracting the performance of the pure binder (or mastic without specific additives) from the composite RAM system and normalizing it, the CR isolates the specific mechanical reinforcement and thermodynamic contribution provided by the filler particles. It should be noted that the CR method assumes a quasi-linear superimposition of effects, which may not fully capture the complex physicochemical interactions in the colloidal system. For instance, at high rejuvenator dosages, the excessive softening of the binder may alter the dispersion state of the filler, leading to non-linear interaction effects that the simplified CR calculation may underestimate. Additionally, uncertainties inherent in contact angle measurements (SFE parameters) can propagate through the CR calculation, potentially introducing statistical noise in the adhesion work contribution analysis. The mathematical formulation of the CR is presented in Equation (7):

(7) $$CR=\frac{Adhesion\;\;index_{RAM}-Adhesion\;\;index_{RA}}{Adhesion\;\;index_{RA}}$$

**Figure 8:**
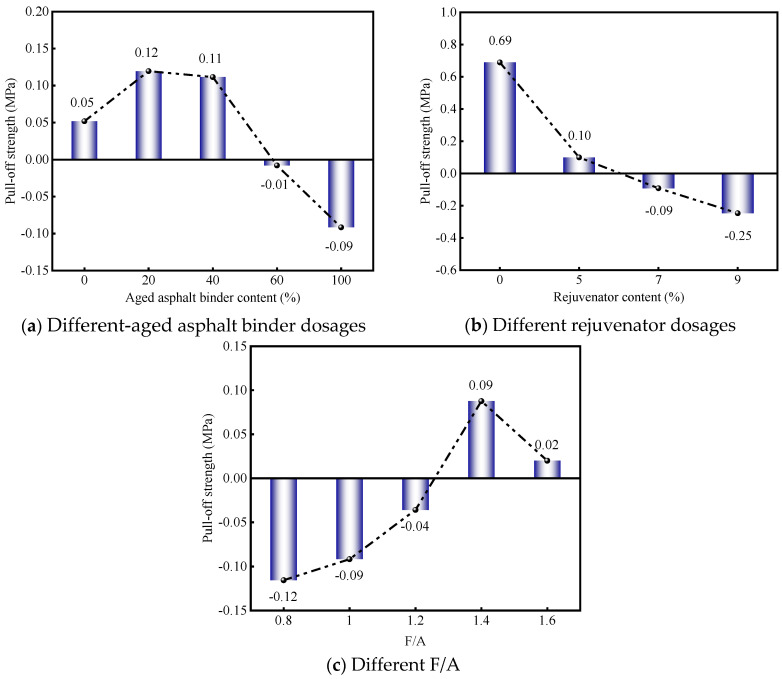
CR of mineral filler to the bonding strength of RAM.

Within this framework, a positive CR value signifies that the mineral filler actively enhances the interfacial adhesion performance, whereas a negative value denotes a reduction in adhesive efficacy attributable to the filler. Figure 8 delineates the CR results concerning the mineral filler’s impact on the interfacial bonding strength. A detailed examination of Figure 8a reveals an inverse correlation between the CR and the content of aged asphalt binder; specifically, the CR diminishes as the dosage of aged binder escalates. Benchmarked against the virgin asphalt mastic, the formulations containing lower-aged binder contents—namely PAV + 20% and PAV + 40%—registered CR increments of 0.07 and 0.06, respectively. Conversely, at higher concentrations, the PAV + 60% and PAV + 100% samples suffered declines of 0.06 and 0.14. These findings elucidate that mineral fillers act as effective reinforcement agents for RAM bonding strength, primarily in systems with low-aged binder dosages. However, in the presence of high-aged binder concentrations, the filler exerts a detrimental influence on bonding integrity.

Data drawn from Figure 8b underscores that the mineral filler achieves its zenith in terms of CR to bonding strength within the aged asphalt mastic matrix. However, the introduction of a 5% rejuvenator dosage precipitated a marked decline in this metric, with the CR dropping by 0.59 relative to the aged mastic baseline. This reduction implies that the presence of rejuvenators partially compromises the structural reinforcement typically provided by the filler. Crucially, as the rejuvenator concentration was further elevated to 7% and 9%, the CR values transitioned into negative territory, revealing that under these conditions, the mineral filler exerts a detrimental rather than beneficial influence on the RAM’s bonding integrity. In essence, the reinforcing potential of the mineral filler is effectively overshadowed by the dominant physicochemical effects of the rejuvenator on the bonding strength.

As illustrated in Figure 8c, the CR remains negative at lower F/A ratios of 0.8, 1.0, and 1.2. This observation indicates that insufficient mineral filler dosage fails to enhance the bonding strength of the RAM. The CR reaches its maximum in the FJB + 1.4 sample, exceeding the value at F/A 0.8 by 0.21. However, upon further increasing the F/A ratio to 1.6, the CR declines by 0.07 relative to the peak. In summary, the capacity of mineral filler to enhance the bonding strength of RAM is finite; specifically, low dosages of mineral filler exert a detrimental effect on the bonding integrity.

#### 3.3.2. The CR of Mineral Filler to Adhesion Work

**Figure 9:**
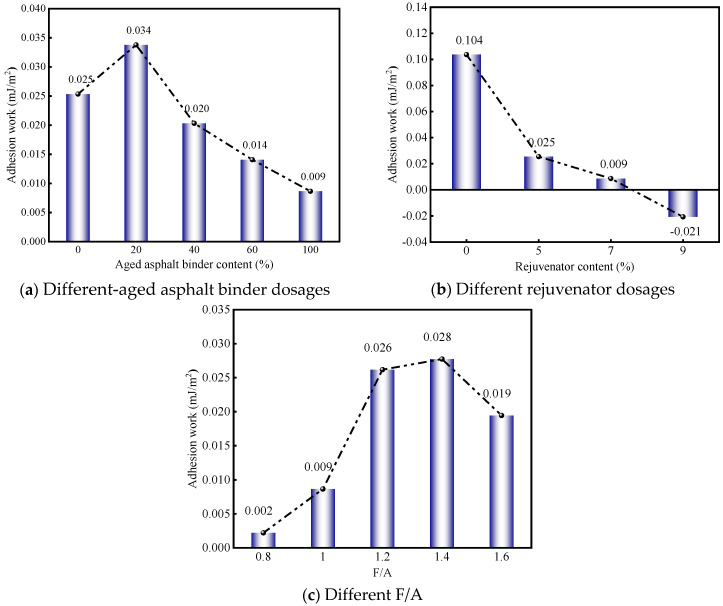
CR of the mineral filler to the adhesion work of the RAM.

Figure 9 illustrates the contribution ratio (CR) of mineral fillers to the adhesion work at the RAM–limestone filler interface. As depicted in Figure 9a, the CR values remain consistently positive across all tested aged binder concentrations. This indicates that the incorporation of mineral filler universally enhances interfacial adhesion work, although the magnitude of this enhancement is modulated by the specific aged binder content. Notably, the PAV + 20% formulation exhibited the highest efficiency, achieving a CR increment of 0.009 relative to the virgin asphalt mastic. This suggests that a moderate inclusion of aged binder acts synergistically to amplify the filler’s positive impact on adhesion performance. However, as the aged binder concentration exceeds this optimal threshold, the CR gradually diminishes. Specifically, relative to the peak observed at PAV + 20%, the CR values for PAV + 40%, PAV + 60%, and PAV + 100% declined by 0.014, 0.020, and 0.025, respectively. Consequently, it can be inferred that the efficacy of mineral filler in enhancing interfacial adhesion is maximized when the aged binder content is maintained at low-to-moderate levels.

Based on the data presented in Figure 9b, the mineral filler initially demonstrates a positive CR of 0.104 within the aged asphalt mastic–aggregate interface system. However, the introduction of rejuvenators significantly alters this dynamic. Specifically, at rejuvenator concentrations of 5% and 7%, the CR values experienced substantial reductions of 0.079 and 0.095, respectively. This downward trend suggests that the presence of rejuvenators tends to attenuate the reinforcing capacity that mineral fillers typically offer to interfacial adhesion work. Crucially, when the rejuvenator content was elevated to 9%, the CR plummeted by 0.125 relative to the aged mastic baseline, resulting in a negative value. This indicates that under high rejuvenator conditions, the mineral filler actually compromises, rather than enhances, the adhesion work at the RAM–limestone filler interface. Consequently, it is evident that the filler’s efficacy is inversely modulated by rejuvenator concentration. To maintain optimal adhesion performance during the binder regeneration process, it is therefore critical to avoid excessive use of rejuvenators.

Figure 9c reveals that, regardless of the specific F/A ratio applied, the CR regarding the adhesion work at the RAM–limestone filler interface consistently remains positive. This implies that within the experimental parameters of this study, mineral fillers invariably exert a beneficial influence on interfacial adhesion, although the magnitude of this benefit is modulated by the F/A ratio. Specifically, at lower F/A ratios of 0.8 and 1.0, the recorded CR values were marginal, standing at 0.002 and 0.009, respectively. These figures suggest that while low filler concentrations do aid adhesion work, the extent of this enhancement is relatively constrained. In contrast, elevating the F/A ratio to 1.2 and 1.4 resulted in more substantial improvements, with CR values rising by 0.024 and 0.026 relative to the 0.8 baseline. However, upon reaching an F/A ratio of 1.6, the CR trajectory reversed and began to decline. This observation confirms that the capacity of mineral fillers to augment interfacial adhesion performance is finite and is strictly governed by the filler dosage.

### 3.4. Multivariate Regression Analysis

Previous analysis indicates that the dosage of aged asphalt binder, the dosage of rejuvenator, and the F/A have a significant effect on the adhesion performance of the RAM–limestone filler interface. To further explore the relationship between these influencing factors and the adhesion performance of the RAM–limestone filler interface, multiple regression analysis was carried out with variables (bonding strength, adhesion work) as dependent variables and various influencing factors as independent variables. While strict multicollinearity checks were not the primary focus, the high determination coefficients (R^2^) and low RMSE values indicate that the model is sufficiently robust for predicting adhesion performance within the defined experimental boundaries. The resulting mathematical model is defined as follows: The multiple regression model can be expressed as follows:

The preceding evaluation has demonstrated that the adhesive properties of the RAM–limestone filler interface are substantially influenced by the content of aged asphalt binder, the concentration of the rejuvenator, and the F/A ratio. In order to systematically quantify the correlations connecting these determinants with adhesion performance, a multiple regression analysis was conducted. Within this statistical framework, the adhesion metrics, namely bonding strength and adhesion work, were designated as the dependent variables, whereas the varying experimental conditions functioned as the independent predictors. The resulting mathematical model is defined as follows:

(8) $$\mathrm{lny}=\alpha +\beta \ln x_{1}+\varphi x_{2}+\lambda x_{3}$$

where *α*, *β*, *φ*, *λ* are regression constants, *y* is the dependent variable, *x_k_* is the aged asphalt binder dosage, rejuvenator dosage, and F/A.

The conjugate gradient method and the general global optimization algorithm are used to fit the curve of the model with 1stOpt. In the 1stOpt software (http://www.7d-soft.com/products.htm, accessed on 5 January 2026), the conjugate gradient method can quickly find the optimal solution in the local range with its fast convergence characteristics, while the universal global optimization algorithm can search for a better solution in the global range. Combining the two can balance the needs of fast convergence and global search, so as to find the optimal solution more effectively.

The best-fit multivariate model in predicting the adhesion performance of the RAM–limestone filler interface is obtained as shown in Table 9. As shown in Table 9, the coefficient of determination R^2^ of the multivariate function model of RAM–limestone filler interface adhesion performance index (bonding strength, adhesion work) and aged asphalt binder dosage, rejuvenator dosage, and F/A is 0.90 and 0.95. RMSE and SSE were 0.0059, 0.0005, and 0.0430, 0.0005, respectively. Indicating that the prediction model is relatively stable, and the error between the predicted value and the actual value is small. In summary, it is credible to use the above model to predict the adhesion performance of the RAM–limestone filler interface; the model has good accuracy.

## 4. Conclusions

In this study, the interfacial adhesion between RAM and aggregates was comprehensively evaluated using pull-off testing and SFE theory. The investigation assessed the impacts of varying aged binder concentrations, rejuvenator dosages, and F/A ratios, while also clarifying the specific CR of mineral fillers. The principal conclusions are as follows:

(1) The bonding strength of the interface follows a parabolic trend, initially rising and then falling as the aged binder content increases. The inclusion of rejuvenators was found to diminish this strength. Furthermore, at low F/A ratios (0.8, 1.0, 1.2), the bonding strength remains below that of the RA baseline. An optimal strength is achieved at an F/A ratio of 1.4.
(2) High concentrations of aged binder were observed to reduce interfacial adhesion work. While adhesion performance surpasses the RA–aggregate interface at rejuvenator dosages of 5% and 7%, higher dosages prove detrimental. Consistent with bonding strength results, both cohesion and adhesion work peak at an F/A ratio of 1.4.
(3) The mineral filler’s CR to adhesion is maximized when the aged binder content is 20%. Increasing the quantity of the rejuvenator tends to attenuate the positive effects of the filler. Similarly, the CR is highest at an F/A ratio of 1.4.
(4) Two multivariate regression models were established to predict bonding strength and adhesion work, achieving R^2^ values of 0.90 and 0.95, respectively. These models demonstrate high accuracy in forecasting the adhesion performance of the RAM–limestone filler interface.
(5) From a practical engineering perspective, the results suggest that when utilizing high dosages of rejuvenators exceeding 7%, the filler-to-asphalt ratio should be moderately increased to approximately 1.4 to compensate for the loss of cohesive strength, thereby ensuring a balanced adhesion performance in recycled asphalt mixtures.

## Figures and Tables

**Table 1:** Physical attributes of the virgin asphalt binder.

Test Items		Value	Specification
Penetration (25 °C,100 g, 5 s), 0.1 mm		87.2	80~100
Ductility (5 cm/min, 10 °C), cm		>100	≥100
Softening point (R&B), °C		47.4	≥42
Film oven aging residues (163 °C, 85 min)	Loss of quality, %	−0.12	−0.8–+0.8
	Penetration ratio (25 °C), %	61	≥54
	Ductility (10 °C), cm	8	≥6

**Table 2:** Physical characteristics of the synthesized rejuvenator.

Test Items	Viscosity (60 °C)	Density	Flash Point	Saturates	Aromatics	Film Oven Aging Mass Loss
	mPa·s	g/cm^3^	°C	%	%	%
Value	103	0.938	248	37.9	46.2	2.6

**Table 3:** Physical specifications of the limestone mineral filler.

Test Items		Value	Specification
Apparent density		2.75	≥2.50
Particle size range	<0.6 mm, %	100	90~100
	<0.3 mm, %	100	75~100
	<0.075 mm, %	78.92	78.92
Plasticity index		3	<4
Hydrophilic coefficient		0.792	<1

**Table 4:** Types of RAM.

Sample ID	Long-Term Aged Asphalt Binder (%)	Virgin Asphalt Binder (%)	Rejuvenator (%)	F/A
ZH	0	100	0	0
PAV	100	0	0	0.0
PAV + 0	0	100	0	1.0
PAV + 20%	20	80	7	1.0
PAV + 40%	40	60	7	1.0
PAV + 60%	60	40	7	1.0
PAV + 100%	100	0	7	1.0
ZSJ + 0	100	0	0	1.0
ZSJ + 5%	100	0	5	1.0
ZSJ + 7%	100	0	7	1.0
ZSJ + 9%	100	0	9	1.0
FJB + 0	100	0	7	0
FJB + 0.8	100	0	7	0.8
FJB + 1.0	100	0	7	1.0
FJB + 1.2	100	0	7	1.2
FJB + 1.4	100	0	7	1.4
FJB + 1.6	100	0	7	1.6

Note: The sample codes are defined as follows: ZH refers to the Zhenhai virgin binder; PAV indicates the long-term aged asphalt binder; ZSJ denotes the rejuvenator and aged asphalt binder; and FJB represents the long-term aged asphalt binder, rejuvenator, and filler-to-binder ratio.

**Table 5:** SFE parameters of the probe liquids (mJ/m^2^).

Liquid	$\gamma_{l}$	$\gamma_{l}^{LW}$	$\gamma_{l}^{+}$	$\gamma_{l}^{-}$
Distilled water	72.8	21.8	25.5	25.5
Glycerol	64.0	34.0	3.92	57.4
Formamide	58.0	39.0	2.28	39.6

**Table 6:** SFE components of the aggregate (mJ/m^2^).

Aggregate	$\gamma_{s}$	$\gamma_{s}^{LW}$	$\gamma_{s}^{AB}$	$\gamma_{s}^{+}$	$\gamma_{s}^{-}$
Limestone	33.59	22.55	11.04	1.49	20.45

**Table 7:** Contact angles between test liquids and RAM (25 °C).

Type of Ram	Distilled Water		Glycerol		Formamide	
	Contact Angle (°)	Variation Coefficient (%)	Contact Angle (°)	Variation Coefficient (%)	Contact Angle (°)	Variation Coefficient (%)
ZH	91.38	0.74	90.12	0.57	87.97	0.51
ZH + PAV	87.75	1.36	84.18	0.91	84.97	0.58
PAV + 0	96.46	0.47	90.52	0.27	87.62	1.10
PAV + 20%	96.97	0.45	90.38	0.81	87.14	1.09
PAV + 40%	95.48	1.16	90.93	0.75	88.01	0.28
PAV + 60%	95.15	0.74	89.88	0.25	88.13	1.59
PAV + 100%	95.74	0.40	89.07	0.74	88.38	0.33
ZSJ + 0	91.86	1.65	89.39	0.62	83.75	1.39
ZSJ + 5%	95.48	1.13	90.33	1.25	87.63	1.23
ZSJ + 7%	95.74	0.40	89.07	0.74	88.38	0.33
ZSJ + 9%	96.97	0.95	89.84	0.23	89.75	1.09
FJB + 0	95.16	0.44	88.37	0.46	88.52	0.39
FJB + 0.8	95.90	0.28	88.49	0.45	88.39	0.30
FJB + 1.0	95.74	0.40	89.07	0.74	88.38	0.33
FJB + 1.2	95.60	0.94	88.09	0.23	87.13	0.26
FJB + 1.4	94.16	0.08	88.51	0.49	87.22	0.46
FJB + 1.6	93.91	0.18	89.91	0.28	87.94	0.65

**Table 8:** The SFE and its components of RAM (mJ/m^2^).

Type of RAM	$\gamma^{LW}$	$\gamma^{+}$	$\gamma^{-}$	$\gamma^{AB}$	γ
ZH	7.89	1.46	10.42	7.81	15.70
ZH + PAV	3.79	3.79	11.80	15.50	19.29
PAV + 0	8.92	1.61	5.61	6.00	14.93
PAV + 20%	9.62	1.50	5.03	5.49	15.10
PAV + 40%	9.00	1.38	6.61	6.04	15.04
PAV + 60%	6.82	2.38	6.78	8.03	14.85
PAV + 100%	4.74	3.69	6.53	9.81	14.55
ZSJ + 0	16.41	0.17	7.91	2.34	18.75
ZSJ + 5%	8.61	1.64	6.38	6.47	15.08
ZSJ + 7%	4.74	3.69	6.53	9.81	14.55
ZSJ + 9%	4.00	4.26	5.86	10.00	13.99
FJB + 0	3.88	4.63	6.69	11.13	15.01
FJB + 0.8	4.18	4.49	6.04	10.42	14.60
FJB + 1.0	5.04	3.72	6.25	9.65	14.69
FJB + 1.2	5.58	3.68	5.77	9.22	14.80
FJB + 1.4	6.21	2.96	7.08	9.16	15.37
FJB + 1.6	7.34	2.00	7.83	7.91	15.25

**Table 9:** Regression results of adhesion performance for RAM–limestone filler interface.

Adhesion Index	Regression Models	Root Mean Square Error (RMSE)	Square Sum of Error (SSE)	R^2^
Bonding strength	$lny_{1}=1.96-0.12lnx_{1}-0.084x_{2}+0.039x_{3}$	0.0059	0.0430	0.90
Adhesion work	$lny_{2}=3.9356-0.013lnx_{1}-0.0125x_{2}+0.017x_{3}$	0.0005	0.0005	0.95

## Data Availability

The original contributions presented in this study are included in the article. Further inquiries can be directed to the corresponding authors.
